# Integrated Cognitive Architecture for Robot Learning of Action and Language

**DOI:** 10.3389/frobt.2019.00131

**Published:** 2019-11-29

**Authors:** Kazuki Miyazawa, Takato Horii, Tatsuya Aoki, Takayuki Nagai

**Affiliations:** ^1^Graduate School of Engineering Science, Osaka University, Osaka, Japan; ^2^Graduate School of Informatics and Engineering, The University of Electro-Communications, Tokyo, Japan; ^3^Artificial Intelligence Exploration Research Center, The University of Electro-Communications, Tokyo, Japan

**Keywords:** cognitive architecture, generative model, concept formation, multimodal categorization, reinforcement learning, language learning, system integration

## Abstract

The manner in which humans learn, plan, and decide actions is a very compelling subject. Moreover, the mechanism behind high-level cognitive functions, such as action planning, language understanding, and logical thinking, has not yet been fully implemented in robotics. In this paper, we propose a framework for the simultaneously comprehension of concepts, actions, and language as a first step toward this goal. This can be achieved by integrating various cognitive modules and leveraging mainly multimodal categorization by using multilayered multimodal latent Dirichlet allocation (mMLDA). The integration of reinforcement learning and mMLDA enables actions based on understanding. Furthermore, the mMLDA, in conjunction with grammar learning and based on the Bayesian hidden Markov model (BHMM), allows the robot to verbalize its own actions and understand user utterances. We verify the potential of the proposed architecture through experiments using a real robot.

## 1. Introduction

The technology of artificial intelligence (AI) in recent years has developed rapidly, exceeding human ability in specific tasks (Russakovsky et al., 2015). Meanwhile, research on artificial general intelligence (AGI) has attracted significant attention (Goertzel, 2014). However, the realization of flexible and versatile intelligence, such as that evident in humans, remains a difficult problem. In particular, it is fair to say that robots that can use language and appropriately plan and perform various actions is not yet a reality. One reason seems to be the lack of physical body in AI. We reason that the physical body is very important in the development of human-like intelligence (Cangelosi and Schlesinger, 2015). This fact is a strong motivation for the premise of using robots in this research.

Another and more important problem is the integration of multiple cognitive modules, which has not been sufficiently studied up to now. Basically, various functions are required for human-like flexible intelligence. Several cognitive functionalities, such as perception, language, and decision making, are intertwined in a complex manner to realize such intelligence. Each of these is often studied independently as a function or algorithmic realization. However, it is important to consider simultaneous learning, involving the overall structure rather than individual elements. Based on such a premise, the research questions seem to be two-fold; how do individual cognitive models relate each to other, and how do they develop each other? In other words, the appropriate connection of all modules should be studied, observing how each module learns in the entire structure. By challenging these questions, we believe we can make a step forward toward realizing AGI by robots. Moreover, from the constructive approach, it is also important to elucidate this core mechanism of human intelligence (Asada et al., 2009).

To pursue the above questions, we first propose a general structure of human-like intelligence at the computational level of the three levels of Marr (1982). The idea of the general structure of human-like intelligence in this study is based on the global network, which has been proposed by Doya (1999). According to Doya (1999), the cerebral cortex utilizes unsupervised learning, and reinforcement learning is carried out in the basal ganglia. Considering the anatomical connection between the cortex and the basal ganglia, our model connects the multi-layered multimodal latent Dirichlet allocation (mMLDA), which implements unsupervised learning, and a reinforcement learning module as the basal ganglia. Multimodal latent Dirichlet allocation (MLDA) is a probabilistic generative model in which LDA is extended to multimodal data, and it is shown that concept learning is possible by applying MLDA to robots (Nakamura et al., 2009; Araki et al., 2012). mMLDA is a model that realizes representation learning of multimodal data and further integrates them hierarchically (Fadlil et al., 2013).

In the proposed model, we combine the Bayesian hidden Markov model (BHMM) with mMLDA. Language learning is realized by combining BHMM, which learns grammar, with mMLDA. This is because the language area corresponds to a module of temporal learning, and the BHMM is used to replicate this functionality. Then, we implement the entire structure and test it using a real robot to reveal the learning process inside. The experiment carried out in this study is based on a scenario of infant-mother interaction. The robot, in which our proposed integrated cognitive model is implemented, interacts with a human partner using some toys. Through the interaction the robot learns object concepts, appropriate actions, and language simultaneously. After the interaction, the model is analyzed to reveal the learning process of the proposed integrated model.

The remainder of this paper is organized as follows. In section 2, we propose the general entire structure of the integrated cognitive model followed by details of each cognitive module in section 3. Section 4 describes the experimental settings and the results of the experiments are explained in section 5. Section 6 discusses the results and section 7 summarizes this paper.

### 1.1. Related Works

Related works include many studies on deep learning. Recent natural language processing has been successful in integrating visual information with natural language. In fact, the system can verbally describe what is contained in the pictures (Arandjelovic and Zisserman, 2017). However, such systems do not address the acquisition of language. Since a supervised learning method is utilized, huge pairs of images and sentences are required to train the system. Furthermore, the language is not grounded to physical real objects nor to actions, since the physical body is not involved. Hill et al. (2017) proposed a neural network-based language learning agent. Although they show promising results, the study was carried out in a simulated 3D world. Hence, it is not clear whether language acquisition can be realized by the interaction between a real robot and a human partner.

The reinforcement learning framework has attracted much attention in recent years, owing to the success of deep Q-networks (DQN) (Mnih et al., 2015). For robotics applications, direct policy search has been used (Levine et al., 2016). Levine et al. (2016) showed the usefulness of reinforcement learning, based on deep learning, in various robotic tasks. Moreover, DQN is also used for manipulation learning (Gu et al., 2017). These studies use real robots to carry out real physical tasks. However, language is not taken into consideration. Although the development of deep learning techniques is remarkable in this way, it focuses on a specific function, and it is difficult to fully understand the inside of the learning process. Furthermore, simulation environments are often used in deep learning research in general.

On the other hand, developmental robotics is a research area that emphasizes the physical body (Cangelosi and Schlesinger, 2015). In the context of developmental robotics, various aspects of development, such as language learning (Morse and Cangelosi, 2017), motor learning (Billard, 2000; Demiris and Khadhouri, 2006), and affordance learning (Stoytchev, 2008; Jamone et al., 2018) are realized using robots. However, there are many studies focusing on individual functions, and few have dealt with concepts, and action learning, and language in an integrative manner.

Tani and Ogata used recurrent neural networks (RNNs) to develop human-like intelligent robots (Ogata et al., 2005; Tani, 2016). Although they showed many interesting results using real robots, it is still an open problem to implement an integrated cognitive model covering areas from the sensor-motor loop to language learning and decision making. In (Heinrich and Wermter, 2018), a cognitive model that is capable of learning language production grounded in both temporal dynamic somatosensation and vision has been proposed. The model is based on multi-timescale RNNs and has properties of hierarchical concept abstraction, concept decomposition, multi-modal integration, and self-organization of latent representations. They also showed some interesting results using a real robot; however, the framework regarding decision making is not involved in the model. Moreover, the RNNs have a common difficulty in the analysis of the model inside, in general.

From the viewpoint of “cognitive architecture,” there are several well-known architectures such as SOAR (Laird, 2012) and ACT-R (Anderson, 2009). In particular, ACT-R is a very famous framework based on cognitive science. There are several cognitive architectures other than SOAR and ACT-R (Kotseruba and Tsotsos, 2018). We consider our proposed model from four viewpoints, which have been mentioned in the study of Kotseruba and Tsotsos (2018). (1) Classification of the cognitive architecture type: our proposed model is classified as an emergent approach because the concepts emerge from our model using sensorimotor information. (2) Types of input modality: our model uses four modalities, namely image, action, language, and reward. (3) Types of cognitive function: we focus on concept formation, decision making, language learning, and integration of each cognitive function. Our model does not consider attention, planning, memory, reasoning, and metacognition, which are often treated in cognitive architectures. (4) Structure of cognitive architecture: our proposed model mainly consists of a combination of probabilistic generative models. The most important point of our model is that it is based on the generative model. The importance of the generative model in intelligence has long been recognized; nevertheless, such cognitive architecture has not been developed. We attribute this to the historical fact that the development of a large-scale probabilistic generative model is complex and technically difficult. Researchers have enabled the development of a large-scale probabilistic generative model by building a distributed learning framework, referred to as SERKET (Nakamura et al., 2018). In addition, a probabilistic programming language has started gaining popularity (Tran et al., 2016); there is a possibility that these aforementioned ideas will be further developed in the future. On the other hand, regarding cognitive architectures for robotics, iCub (Vernon et al., 2011) and ISAC (Gordon et al., 2010) exist. However, the structure of each of these architectures is different from that of our proposed model. Essentially, they follow the structure of conventional cognitive architectures.

One of the promising research directions toward AGI is the use of generative probabilistic modeling (Taniguchi et al., 2016b). In fact, some studies have shown the usefulness of the probabilistic models to acquire knowledge by self-organizing multimodal information that the robot obtains through its own experience. Because such knowledge is abstracted and linked to language, it can be reused for various tasks. Until now, there have been few cases in which action learning through reinforcement learning, concept acquisition, and language learning have been handled in a unified manner. In the previous models including (Nishihara et al., 2017), probabilistic representations of knowledge has been proposed; however, the mechanisms of decision making have not been involved. It is important to reveal how robots experience and collect multimodal data in the first place. Furthermore, how do the robots use the acquired knowledge to decide their own actions? Answering these questions is necessary for the integration of the frameworks of concept formation, language acquisition, real-world understanding, and action planning from motion learning by trial and error.

By contrast, to create complex human-like intelligence that operates in the real world, it is necessary to consider complex cognitive functional units that work together. Thus, in our recent research, we proposed a framework to integrate multiple modules (Miyazawa et al., 2017). However, we were limited to the integration of concept formation and reinforcement learning. Therefore, in this study, we further develop and propose a model to simultaneously learn concepts, language, and actions. We propose a framework to realize concept learning, knowledge acquisition, language learning, and decision making by integrating various modules, mainly the mMLDA. We verify the potential of this proposed framework through experiments in a real environment using a real robot.The main contribution of this research is that a unified framework for realizing such actions and language acquisition loop is assembled around the mMLDA. Decision making, and language understanding, using abstracted concepts, are verified using a real robot.

## 2. Integrated Cognitive Architecture

In this section, we propose a general framework of integrated cognitive architecture.

### 2.1. Framework

The idea behind our proposed integrated architecture is based on the hypotheses in Doya (1999), which claim that the cerebral cortex is used for unsupervised learning, the basal ganglia is for reinforcement learning, and the cerebellum is for supervised learning. This hypothesis implies that the unsupervised learning module is a core for the entire system to integrate multimodal sensor motor signals. In other words, the unsupervised learning module works as a learning representation of hierarchically integrated multimodal sensor-motor signals, which generates a latent space.

The temporal learning module is connected to the central unsupervised learning module through the latent space. The temporal module correspond to the grammar learning module that encodes and decodes utterances. In the brain, this module corresponds to the language area. From the anatomical viewpoint, the basal ganglia forms some loops with the cortex. This motivates us to connect the unsupervised learning module to a reinforcement learning module through the latent space.

**Figure 1:**
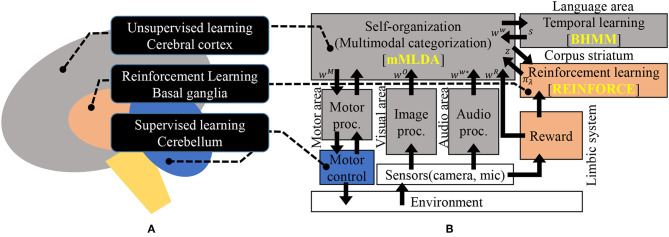
An overview of the proposed integrated model; **(A)** illustration of the hypothesis in Doya (1999), and **(B)** the proposed architecture corresponding to **(A)**. Please refer to Figure 3 for notation. Figure 3 is a proposed implementation of **(B)** using probabilistic generative models.

Figure 1 illustrates the idea of our proposed integrated cognitive model. The hypothesis in (Doya, 1999) is depicted in Figure 1A. Figure 1B represents the corresponding model of Figure 1A. The important point of this model is that it exhibits a hub-like structure based on probabilistic generative models.In other words, it is a framework in which various modules can be connected via latent variables. In Figure 1B, the algorithms that were used to implement the modules in this study are indicated with yellow characters. The MLDA is used for multi-modal categorization, the BHMM is used for time-series learning in the language area, and the REINFORCE algorithm is used for reinforcement learning. These are the main modules in the proposed model and their details will be described in section 3. The remaining modules, which mainly perform data acquisition and preprocessing, will be described in section 3.1.1.

In this study, the minimum modules necessary for autonomous learning of robots are integrated; however, many other modules can be connected in the same manner. Another important point to note is that with the proposed structure, the entire model can be optimized using the SERKET framework (Nakamura et al., 2018) described later.

### 2.2. mMLDA as Unsupervised Learning Module

**Figure 2:**
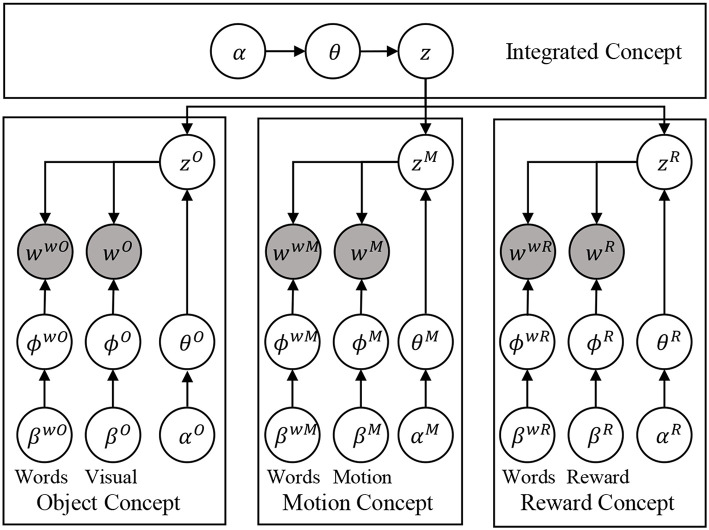
The graphical model of the mMLDA used in this paper for formed concepts. Please refer to Figure 3 for notation.

According to the above discussion, first, an actual algorithm is selected for the unsupervised learning module. We select MLDA as it has been proven that the real robots can form abstract concepts through unsupervised learning by using MLDA (Nakamura et al., 2009). Since a hierarchical structure is required in the integration, we use hierarchical version of MLDA, which is called multi-layered MLDA (mMLDA) (Fadlil et al., 2013; Attamimi et al., 2016). The mMLDA routine stochastically expresses multiple concepts and their relationships as shown in Figure 2.

Now, validity of the choice of mMLDA in this study is discussed. There are three good reasons for the use of mMLDA in this task. First of all, as mentioned above, there are some successful robotics applications of MLDA (Araki et al., 2013). The second reason is the hypothesis that, as shown in (Tomasello et al., 2017), the integration of multimodal information in the cortex is achieved by the bidirectional connection of each area. Of course, we are not claiming that the mMLDA expresses the structure of the brain as it is, but it is suggested that it imitates the functional aspect of the brain in the integration of multimodal information. The last reason is the hub structure revealed by Connectome (Hagmann et al., 2008). The mMLDA part in Figure 2 has a hub structure centered on latent variables, and functional similarity with the brain is seen in this respect.

### 2.3. Whole Structure

The proposed model consists mainly of mMLDA and a combination of several modules. The central role of mMLDA is to form concepts by categorizing sensorimotor information, as discussed above. The modules, which utilize formed concepts, consist of the BHMM, which deals with language, and reinforcement learning for decision making. Again, the important idea behind our proposed model is the use of latent variables as the connectors of several modules. It is worth noting that mMLDA shown in Figure 2 has a structure based on concepts such as objects, motions, and reward rather than the input modalities directory. These concepts are the minimum necessary concepts when the robot acts on its own and learns language through trial and error, and through interaction with human partners. Of course, there are other concepts as well. For example, spatial concepts (Taniguchi et al., 2016a) can be considered; however, to realize such a concept, the robot is required to have a mobile base.

### 2.4. Language Module

In this model, word information is grounded in real-world information through mMLDA. The lexicon is represented by the word nodes in Figure 2, which corresponds to the audio processing part in Figure 1B. By applying syntactic information encoded in the BHMM to this word information, this model can generate sentences. Conversely, by decomposing sentences using the syntax encoded in the BHMM, followed by the prediction of real-world information, the robot can understand the meaning of sentences. The BHMM is used for the implementation, since the BHMM has been shown to be capable of learning linguistic structures in an unsupervised manner (Goldwater and Griffiths, 2007).

### 2.5. Decision Making/Action Module

Regarding decision making, we consider instantaneous decision making using a learned policy function. Therefore, the proposed model integrates a reinforcement learning algorithm. The most important aspect of this learning action is that the latent variable defined in the mMLDA is used as the state space for the reinforcement learning. Since the learning of the model is carried out simultaneously, the representation of the mMLDA is affected by both sensorimotor signals and the policy function learned by the reinforcement learning module. Furthermore, language also affects the learning of actions.

In the proposed model, discrete actions are assumed, which are encoded in the motor processing in Figure 1B. This indicates that the reinforcement learning module learns to select an appropriate discrete action to maximize the total reward. Although the discrete actions are assumed to be fixed throughout the learning in this study, an action learning method can be involved in the motor processing. The reward can be the primary reward and/or secondary one; however, the mechanism must be designed some way. Moreover, reward signals are input to the mMLDA to categorize and connect to the word information. This makes it possible for the robot to ground the meaning of words such as “good” and “bad.”

### 2.6. Limitations

A limitation of this architecture is the lack of a temporal learning module. Without the temporal learning module, it is impossible for the robot to plan long-term behavior. In fact, the learning of temporal patterns in PFC is an important functionality of the cortex (Shima et al., 2007). Although the current model does not have a temporal learning module, it is possible for the model to add another BHMM as a temporal learning module. By using time series information expressed in the BHMM, dynamic programming such as the Viterbi algorithm, makes it possible to plan long-term action sequences toward a specified goal state. Furthermore, by combining immediate and long-term actions through the subsumption architecture (Brooks, 1991), the model enables a wide variety of behavior for the robot.

Another important limitation to note is that of the structure of the model. Since parametric Bayesian models are involved in the proposed model, the number of classes must be defined in advance. This causes a serious problem when aiming for open-ended learning by the robot. However, this can be overcome by applying Bayesian non-parametric models. Furthermore, what type of modules are required for achieving human-like intelligence is an important question that should be pursued in the future.

From the viewpoint of constructive approach, the proposed model also has a limitation, that it cannot be directly compared with human cognitive functions because it has not been designed to verify them. More specifically, each sensor input has been simplified and is likely to deviate from human perception. For example, information regarding motion is used as input in a discrete manner; thus, the learning of motion concepts is a simple task compared with human learning. This approach does not reflect the difficulties that humans face with regard to language learning, such as difficulties in learning nouns and verbs.

## 3. Implementation of Modules

**Figure 3:**
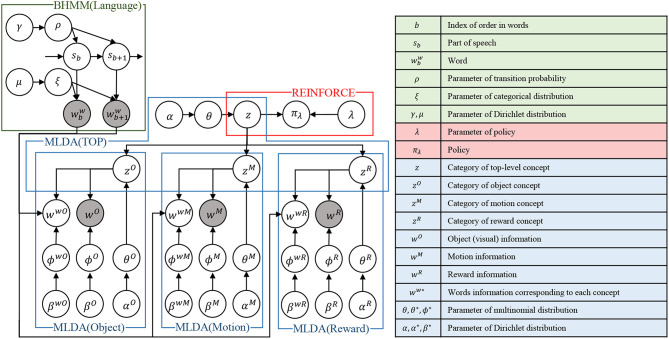
An overview of the proposed integrated cognitive model. Notations are listed in the right table. It should be noted that time index *t* is omitted in this figure for simplicity. The top-level concept at time *t* is fully represented as *z*_*t*_ instead of *z* for example.

Figure 3 shows the overall structure of the proposed integrated cognitive model, which is realized by combining each module. We describe the details of concept formation, language learning, and policy learning in the next section. Then, we describe the learning method of the entire model.

### 3.1. Concept Formation (Unsupervised Representation Learning)

The concept formation uses mMLDA, which stochastically expresses multiple concepts and their relationships. The mMLDA framework exhibits a hierarchical structure with multiple MLDAs that express subordinate concepts, such as objects, and motion, in the lower layer, and an MLDA that integrates them in the upper layer. In this study, we use mMLDA which contains object, motion, and reward concepts. mMLDA allows the categorization of each sensor-motor signal, and also includes simultaneous unsupervised learning of the relationship between these concepts (Attamimi et al., 2016).

The graphical model of the mMLDA used in the experiment is illustrated in Figure 2, in which *z* is a category representing an integrated concept, and *z*^*O*^, *z*^*M*^, and *z*^*R*^ are objects, motion, and reward concepts, respectively, corresponding to subordinate concepts. The top-level concept *z* captures the relationship between lower-level categories and expresses the state of the robot. Variables *w*^*O*^, *w*^*M*^, *w*^*R*^, and *w*^w^* are observations representing object (visual) information, robot motion, reward, and word information, respectively. ϕ^*^ and θ* are parameters of multinomial distributions. β* and α* are parameters of Dirichlet distributions. We briefly explain each observation as follows.

#### 3.1.1. Observed Information

The robot observes multimodal information through various sensors by acting in a real environment. We will explain in detail how the robot acquires information by interacting with the environment in the experiment section.

Object information *w*^*O*^ is extracted from the image captured by the RGBD camera attached to the robot. The object region is segmented out from the acquired image containing multiple objects. From the object region image, feature extraction is performed using AlexNet, which is a convolutional neural network (CNN) pre-trained with ImageNet. Specifically, we use the 4,096-dimensional activation vector of the layer just before AlexNet's final layer when inputting an object image. Since the input of MLDA needs to be a histogram, the obtained 4096-dimensional vector is rounded off, and negative values are replaced with zero.

Next, motion information is explained. In this experiment, the robot motion is performed by selecting one of four manually designed motions. Therefore, motion information *w*^*M*^ is a discrete value, and the 4-dimensional one-of-*k* representation, corresponding to the selected robot motion, is used. Details of each motion of the robot are described in the experiment section.

Reward information *w*^*R*^ uses the reward value obtained according to the robot's action. The reward value is given to the robot's action using direct key input by a person based on the reward function. Since reward information *w*^*R*^ also needs to be expressed as a histogram, reward value *r* is divided into three cases according to *r* < 0, *r* = 0, *r* > 0, and the one-of-*k* representation is used.

Linguistic information *w*^w^* is obtained from human utterances captured by microphone. A speech recognizer converts the acquired speech waveform into sequences of text. Each sentence is divided into words by a morphological analyzer, and the occurrence frequency of each word is calculated to transform the sentence into a bag-of-words representation. Then, for the acquired word information, linguistic information *w*^w^* corresponding to each concept (e.g., object, motion, and reward) is estimated by weighting the word information described later.

#### 3.1.2. Parameter Estimation and Prediction

In this research, inference of mMLDA is realized by inference of multiple MLDAs and message passing in the framework of SERKET (Nakamura et al., 2018) described later. Since the inference is based on the technique for single MLDA, the update rule of MLDA (Nakamura et al., 2009) is briefly explained in Appendix A, and in section 3.4.1, the learning of the entire mMLDA in the SERKET framework is described. It is also possible to estimate concepts (categories) for new data using learned models. Appendix B summarizes the prediction method using the leaned MLDA model.

### 3.2. Language Learning

In language learning, robots learn concepts, grammar, and words mutually through mMLDA and BHMM using the method proposed by Attamimi et al. (2016). In this method, the grammar represents information on the part-of-speech (POS) of each word and syntax. The POS is represented by concept classes, and the syntax expresses the order in which these concept classes are arranged to construct a sentence using the Markov model. Therefore, the problem of POS-tagging comprises the estimation of a conceptual class to express each extracted word. The detailed formulation of language learning is shown in Appendix C.

In contrast, the mMLDA is trained using the provided POS information as the weights for corresponding concepts. Thus, to form concepts by mMLDA, it is important to estimate the POS of each word, which is a part of syntax.

### 3.3. Policy Learning

For policy learning, the REINFORCE algorithm, which is a policy gradient method (Williams, 1992), is utilized. In this study, decision making corresponds to two selections. One is the choice of the motion that the robot should take, and the second is the choice of the object that the robot should handle. Because the top-level concept *z*_*t*_ generates the motion concept $z_{t}^{M}$ and the object concept $z_{t}^{O}$ in the proposed generative model, the action selection is realized by estimating the top-level concept *z*_*t*+1_ at the next time step so that the accumulated reward is maximized (please note that the subscript “*t*” is used as the time index in this section). In other words, integration of mMLDA and reinforcement learning is performed by setting the top-level concept, which is formed by mMLDA, as the state space and actions of reinforcement learning. The actual algorithm of the proposed reinforcement learning using observed multimodal information is described below.

First, the proposed motion and object selection method is explained. The motion selection is performed by sampling the following equation using the top-level concept:

(1) $$\begin{array}{r} {\hat{w}}_{t}^{M}\sim P\left(w_{t}^{M}|{\bar{z}}_{t}\right)=\underset{z_{t}^{M}}{\sum }P\left(w_{t}^{M}|z_{t}^{M}\right)P\left(z_{t}^{M}|{\bar{z}}_{t}\right), \end{array}$$

where ${\bar{z}}_{t}$ represents the top-level concept, which is predicted using the policy described later. Here let the vector **z** be vector representation of the probability distribution *P*(*z*). The object selection is performed by measuring the difference between each object concept of candidate objects and the object concept ${\hat{z}}_{t}^{O}$, which is estimated from ${\bar{z}}_{t}$:

(2) $$\begin{array}{r} {\hat{z}}_{t}^{O}={\left[P\left(z_{t}^{O}=1|{\bar{z}}_{t}\right)P\left(z_{t}^{O}=2|{\bar{z}}_{t}\right)\cdots P\left(z_{t}^{O}=N_{o}|{\bar{z}}_{t}\right)\right]}^{T}, \end{array}$$

where ${\hat{z}}_{t}^{O}$ and *N*_*o*_ denote vector representation of the distribution $P\left(z_{t}^{O}|{\bar{z}}_{t}\right)$ and the number of object categories, respectively. Robots acquire multimodal information $w_{obs,t}=\left[w_{t}^{O}w_{t}^{M}w_{t}^{R}w_{t}^{w*}\right]$ through multiple sensors by performing the selected motion with the selected object. In section 4, information acquisition will be described in detail.

Now, we will describe the calculation of the policy, that is, the estimation of the top-level concept ${\bar{z}}_{t+1}$ at time *t* + 1 from ẑ_*t*_. The distribution of the top-level concept at time *t*, i.e., ${\hat{z}}_{t}$, can be estimated from the observed information ***w***_*obs, t*_ using Gibbs sampling:

(3) $$\begin{array}{r} {\hat{z}}_{t}={\left[P\left(z_{t}=1|w_{obs,t}\right)P\left(z_{t}=2|w_{obs,t}\right)\;\cdots P\left(z_{t}=K|w_{obs,t}\right)\right]}^{T}.\; \end{array}$$

*ẑ*_*t*_ inferred from observations at time *t* is used as the state space for the reinforcement learning. In the proposed method, decision making is to predict the top-level concept ${\bar{z}}_{t+1}$ at the next time step *t* + 1, since the robot motion and the target object can be calculated based on Equations (1) and (2) once ${\bar{z}}_{t+1}$ has been obtained. Therefore, categories *k* of the top-level concept ${\bar{z}}_{t+1}$ can be seen as actions in the reinforcement learning. As Equation (4) shows, we calculate the policy using the estimated top-level concept *ẑ*_*t*_ and softmax function:

(4) $$\begin{array}{r} \pi_{\lambda }\left({\bar{z}}_{t+1}=k|{\hat{z}}_{t}\right)=\frac{exp\left\{\lambda^{T}\psi \left({\bar{z}}_{t+1,k},{\hat{z}}_{t}\right)\right\}}{\sum_{k}exp\left\{\lambda^{T}\psi \left({\bar{z}}_{t+1,k},{\hat{z}}_{t}\right)\right\}}, \end{array}$$

where λ and ${\bar{z}}_{t+1,k}$ denote the parameter vector of the policy function and the one-hot vector whose *k*-th component is one assuming ${\bar{z}}_{t+1}=k$, respectively. ψ(·) calculates the feature vector of size *K*^2^ using Kronecker product of ${\bar{z}}_{t+1,k}$ and ${\hat{z}}_{t}$, where *K* represents the number of top-level categories. Then, the distribution of ${\bar{z}}_{t+1}$ can be obtained by calculating the policy function for all *k*:

(5) $$\begin{array}{l} {\bar{z}}_{t+1}=[\pi_{\lambda }({\bar{z}}_{t+1}=1|{\hat{z}}_{t}),\pi_{\lambda }({\bar{z}}_{t+1}=2|{\hat{z}}_{t}),\cdots ,{\pi_{\lambda }({\bar{z}}_{t+1}=K|{\hat{z}}_{t})]}^{T}. \end{array}$$

At time *t* + 1, new observations ***w***_*obs, t*+1_ are acquired by acting on the basis of the above-mentioned policy. *P*(*z*_*t*+1_|***w***_*obs, t*+1_) can be calculated by mMLDA from the acquired observations ***w***_*obs, t*+1_, and this provides the state ${\hat{z}}_{t+1}$ at time *t* + 1. Hence, at time *t* + 1, the policy is calculated by $\pi_{\lambda }\left({\bar{z}}_{t+2}=k|{\hat{z}}_{t+1}\right)$.

The parameter λ is updated by

(6) $$\begin{array}{rcl} \lambda^{\tau +1} & = & \lambda^{\tau }+\eta \nabla_{\lambda }J\left(\lambda \right), \end{array}$$

(7) $$\begin{array}{rcl} \nabla_{\lambda }J\left(\lambda \right) & \approx & \overset{E}{\underset{e=1}{\sum }}\overset{T}{\underset{t=1}{\sum }}\left(R_{t}^{e}-\bar{b}\right)\nabla_{\lambda }\log\pi_{\lambda }\left({\hat{z}}_{t}^{e}=k_{t}^{e}|{\hat{z}}_{t-1}^{e}\right), \end{array}$$

where η represents the learning rate, $R_{t}^{e}$ is the reward at *t*-th step in *e*-th episode, and $\bar{b}$ denotes mean reward. $k_{t}^{e}$ is the category of the top-level concept obtained by $k_{t}^{e}={\text{argmax}}_{k}{\hat{z}}_{t}^{e}$. *E* and *T* represent respectively the total number of episodes and the total number of steps at each episode for calculating ∇_λ_*J*(λ) during the parameter update process.

### 3.4. Learning and Recognition of Whole Integrated Model

**Figure 4:**
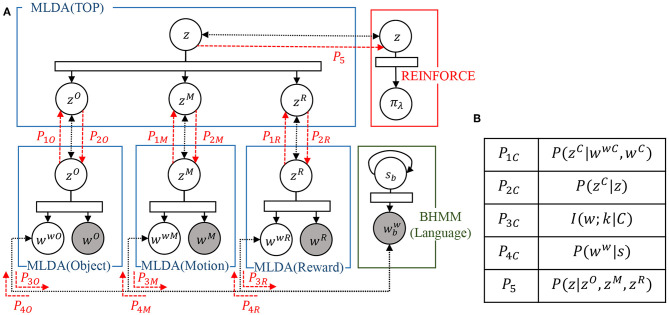
An overview of the proposed integrated model implemented using SERKET framework (Nakamura et al., 2018): **(A)** connection of the modules, and **(B)** messages passed through the connections.

The integration of the models is realized by message passing in the SERKET framework proposed by Nakamura et al. (2018) The proposed integrated cognitive architecture in Figure 3 is implemented as a combination of modules as shown in Figure 4A. Then, the modules are communicated to each other by passing the messages, which are shown in Figure 4B. Please note that Figure 4A omits the description except for important variables. Implementing the entire model through module integration makes it easy to implement and add modules.

#### 3.4.1. Learning of the Whole Model

Here, we describe how to combine each module and the learning method to update the entire model. First, realization of mMLDA by integrating multiple MLDAs is described followed by the integration of mMLDA and BHMM. Finally, the integration of mMLDA and reinforcement learning is described.

mMLDA is realized by integrating multiple MLDAs. This means that *P*(*z*^*C*^|*w*^*wC*^, *w*^*C*^) estimated by the lower layers (concept *C* ∈ {*Object, Motion, Reward*}) and *P*(*z*^*C*^|*z*) estimated by the top layer are exchanged with each other. First, each low-level concept *z*^*C*^ is estimated by MLDA:

(8) $$\begin{array}{l} {\hat{z}}_{j}^{C}=[P(z_{j}^{C}=1|w_{j}^{C},w_{j}^{wC})P(z_{j}^{C}=2|w_{j}^{C},w_{j}^{wC})\cdots {P(z_{j}^{C}=N_{C}|w_{j}^{C},w_{j}^{wC})]}^{T}, \end{array}$$

where $w_{j}^{*}$, ${\hat{z}}_{j}^{C}$, and *N*_*C*_ denotes the *j*-th observation of each modality, each concept allocated to all the *j*-th observation, and the number of each concepts categories. Then, the ${\hat{z}}^{C}$ is sent to the top layer. The parameters of the top layer (MLDA) are updated by sampling the sent low-level concepts as observations using the method in Appendix A (see Equation A-1). Then, the low-level concept is inferred using the top-level concept in the top layer.

(9) $$\begin{array}{r} P\left(z^{C}|{\hat{z}}_{j}^{O},{\hat{z}}_{j}^{M},{\hat{z}}_{j}^{R}\right)=\underset{z}{\sum }P\left(z^{C}|z\right)P\left(z|{\hat{z}}_{j}^{O},{\hat{z}}_{j}^{M},{\hat{z}}_{j}^{R}\right). \end{array}$$

These estimated values are sent to each lower layer. The lower layer uses Gibbs sampling to update parameters using the received message by the following equation:

(10) $$\begin{array}{r} z_{mij}^{C}\sim P\left(z_{mij}^{C}=k|\text{W},{\text{Z}}^{\backslash mij},\alpha ,\beta^{m}\right)P\left(z^{C}|{\hat{z}}_{j}^{O},{\hat{z}}_{j}^{M},{\hat{z}}_{j}^{R}\right), \end{array}$$

where **W** denotes the observed multimodal information. **Z**^\*mij*^ is the remainder set of concepts after removing category *z*_*mij*_ assigned to the *i*-th information of the modality *m* of the *j*-th data. Equation (10) is obtained by multiplying Equation (A-1) in Appendix A by each lower-level concept $P(z^{C}|{\hat{z}}_{j}^{O},{\hat{z}}_{j}^{M},{\hat{z}}_{j}^{R})$ estimated in the top layer. As a result, the lower-level concept and the upper-level concept are updated mutually.

The integration of mMLDA and BHMM is realized by passing the mutual information *I*(*w*; *k*|*C*) of word *w* and category *k* in concept *C*, and the output probability *P*(*w*^*w*^|*s*) of the word *w*^*w*^ output from the POS *s* obtained by the BHMM. The integration of mMLDA and reinforcement learning is realized by passing the concept *z*, which is inferred in the top layer, to use it as the state-space for the reinforcement learning. As a result, the entire model is integrated, and each module performs learning while affecting each other. The procedure for updating and message passing for each of these modules is shown in the **Algorithm 1**. The entire model is updated according to this update rule.

**Algorithm 1:** Learning algorithm of the integrated cognitive model. Please refer to Appendix for Equations A-1 and A-11.

observe *w*^*O*^,*w*^*M*^,*w*^*R*^,*w*^*w*^
set initial random value to *z*,*z*^*O*^,*z*^*M*^,*z*^*R*^,*s*,λ
set *w*^*wC*^←*w*^*w*^
**for** *i*←0 to *n*−1 **do**
update parameter of lower MLDA *z*^*O*^,*z*^*M*^,*z*^*R*^ (See Equation 10)
send *P*_1*C*_ to top MLDA
send *P*_3*C*_ to BHMM
update parameter of top MLDA *z* (See Equation A-1)
send *P*_2*C*_ to lower MLDA
update parameter of BHMM *s* by using *P*_3*C*_ as an initial value (See Equation A-11)
send *P*_4*C*_ to lower MLDA
update word information *w*^*wC*^ (See Equation 11)
**end for**
send *P*_5_ to REINFORCE
update parameter of REINFORCE λ (See Equations 6 and 7)

In addition, in the proposed model, learning can be performed online from data acquired by the framework of reinforcement learning. This can be done by updating each module for each set of data acquired in a certain episode. The latent variables inferred from data acquired before an episode are fixed, and Gibbs sampling are applied only to new data. This enables online model updating.

#### 3.4.2. Various Recognition Using the Model

**Figure 5:**
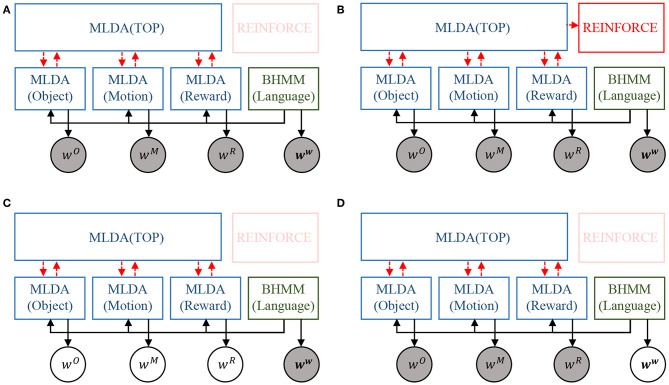
Various recognition tasks using the model: **(A)** category recognition, **(B)** Decision making, **(C)** language understanding, and **(D)** sentence generation. Please note that the random valuables in gray and white nodes represent the observation and the task to be predicted, respectively.

By using the learned model, various cognitive functions such as decision making, language understanding, and sentence generation can be realized. Recognition using the integrated cognitive model is performed by predicting unobserved information. Various recognition tasks using the learned model are shown in Figure 5. The estimation of the category of each concept is equivalent to inferring concepts *z, z*^*O*^, *z*^*M*^, *z*^*R*^ from the observations *w*^*O*^, *w*^*M*^, *w*^*R*^, *w*^*w*^ as shown in Figure 5A. The recognition in the mMDLA is performed by fixing the learned parameters and Gibbs sampling only on the observed information to be recognized, as shown in Appendix B. The estimation of *w*^*wC*^ at recognition is first performed by following equation:

(11) $$\begin{array}{r} w^{wC}\propto w^{w}P\left(w^{w}|s\right), \end{array}$$

where *P*(*w*^*w*^|*s*) represents the output probability of BHMM. For details on *P*(*w*^*w*^|*s*), please refer to Equation (A-10) in Appendix C. Then, using *w*^*O*^, *w*^*wO*^, *w*^*M*^, *w*^*wM*^, *w*^*R*^, and *w*^*wR*^ as observations, the concepts *z, z*^*O*^, *z*^*M*^, and *z*^*R*^ are estimated by updating the low-level concepts and the top-level concept mutually. Because the concept is represented by a mixture of categories, the category *k* of the concept is obtained by selecting the category with the highest probability.

In the action selection, the action of the next step is determined from the observations *w*^*O*^, *w*^*M*^, *w*^*R*^, *w*^*w*^, as shown in Figure 5B. This is done by passing the ${\hat{z}}_{t}$, which is estimated by the language weighting, using BHMM and the mutual updating of each MLAD described above in the estimation of the category, to the reinforcement learning module. By using the passed ${\hat{z}}_{t}$, the reinforcement learning module can estimate the top-level concept ${\bar{z}}_{t+1}$ of the next time step using Equations (4) and (5). By passing the estimated top-level concept ${\bar{z}}_{t+1}$ to the object concept module and the motion concept module, Equations(1) and (2) can be calculated. This makes it possible for the robot to select an object and action to take.

Here, language understanding is defined as the prediction of real-world information *w*^*O*^, *w*^*M*^, *w*^*R*^ for given linguistic information *w*^*w*^, as shown in Figure 5C. This can be done by weighting the linguistic information based on the BHMM in Equation (11) and predicting unobserved information by MLDA. In other words, the linguistic information corresponding to each concept *w*^*wO*^, *w*^*wM*^, *w*^*wR*^ is determined using Equation (11). With this as observation, unobserved real-world information *w*^*O*^, *w*^*M*^, *w*^*R*^ can be obtained by MLDA.

As shown in Figure 5D, the generation of sentence is equivalent to generating a word sequence *S* that is suitable for the observed real-world information *w*^*O*^, *w*^*M*^, *w*^*R*^. This can be done as shown in Appendix D. The important aspect in this sentence generation is that the inference of mMLDA is realized by message passing.

## 4. Experiment

An experiment involving the interaction with the environment and a human partner using a real robot is conducted. In this experiment, the robot learns concepts, language, and actions through trial and error from the state in which the robot has no knowledge of the environment. The purpose of the experiment is to verify the validity of the model by analyzing the results of learning.

### 4.1. Experimental Setup

**Figure 6:**
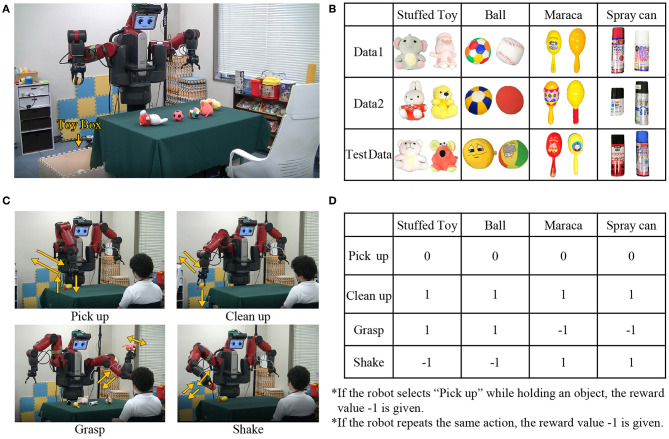
Experimental settings: **(A)** the robot, **(B)** objects, **(C)** designed motions, and **(D)** reward.

The experimental setup is shown in Figure 6. A dual-arm robot (Baxter) shown in Figure 6A is used for the experiment. The robot learns to select one of four types of motions shown in Figure 6C for four types of objects, shown in Figure 6B. A total of 24 objects are used in the experiment, and six sets of stuffed animals, balls, maracas, and spray cans are included in the object set. These objects are divided into three groups as shown in Figure 6B. We use Data 1 and Data 2 for learning and test data for evaluation. The motions shown in Figure 6C is designed in advance by hand. The observed information acquired by the robot and the preprocessing of each piece of information have been described in section 3.1.1. The hyperparameters of each module were set as follows. The number of categories for each concept of the mMLDA was 4 for object concepts, 4 for motion concepts, 3 for reward concepts, and 24 for integrated concepts. The number of dimensions in the state space and the action space for reinforcement learning was 24, and the number of hidden states in the BHMM was 24. Each module was updated 100 times during learning and 20 times during recognition. The number of updates for the entire model, including message passing, was set to 5.

**Figure 7:**
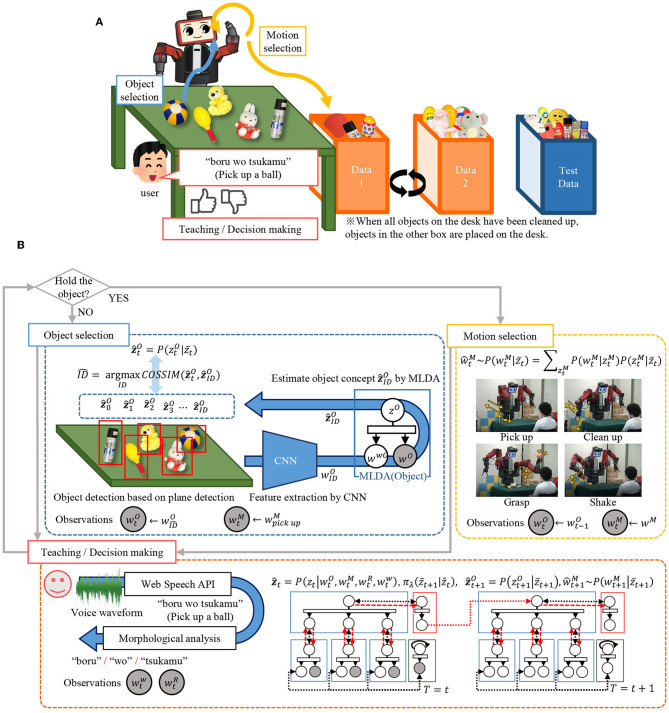
Flow of the experiment: **(A)** procedural overview of the experiment, and **(B)** action selection and acquisition of information by the robot.

Figure 7 shows the overall flow of the experiment, and the action selection and information acquisition by the robot. Now, the outline of the experiment is explained. The robot learns to select an object from the desk and select an appropriate motion, as shown in Figure 7A. When the robot cleans up an object, the objects on the desk are reduced by one. When all the objects are put away, the other object set (Data 1 or 2) is placed on the desk. When the robot acts, the human partner gives verbal information and rewards corresponding to the object and the motion. In this setting, the highest reward can be obtained by repeating a set of motions of grasping an object (“pick-up”), performing the appropriate motion (“grasp” or “shake”), and putting it in the box (“clean-up”). Through this experience, the robot learns concepts, actions, and languages simultaneously.

Next, we will describe the details of decision making and information acquisition. The robot performs action selection and information acquisition according to the flow shown in Figure 7B. The action selection by the robot is classified into two types: object selection and motion selection, depending on whether or not an object is held.

If the robot does not hold an object, object selection is performed. Object selection is performed using the object concept estimated for the object on the desk and that calculated based on its own policy. The robot extracts the planar region of the tabletop using the point cloud acquired by the RGB-D sensor installed on the head and detects objects on the plane as candidates. For each detected object region, feature extraction using convolutional neural network (CNN) is performed to obtain the feature $w_{ID}^{O}$ for each object *ID*. The object concept ${\hat{z}}_{ID}^{O}$ is estimated using MLDA for each $w_{ID}^{O}$. From the top-level concept, ${\bar{z}}_{t}$ is calculated by the policy explained earlier, and ${\hat{z}}_{t}^{O}$ is calculated using Equation (2). The cosine similarities between ${ẑ}_{t}^{O}$ obtained from the top-level concept ${\bar{z}}_{t}$ and ${ẑ}_{ID}^{O}$ are calculated for all the estimated objects. Then, the object with the highest cosine similarity is selected. The object is chosen in this way, and then, the robot acquires $w_{t}^{O}$ and $w_{t}^{M}$.

If the robot is holding an object, it performs motion selection. The motion selection is performed by predicting the motion information $w_{t}^{M}$ from the top-level concept ${\bar{z}}_{t}$ given by the reinforcement learning module according to the Equation (1) When “pick-up” is selected, no motion is undertaken because the object is already held. When “clean-up” is selected, the object is put in a box, and the state is transitioned to the non-grasping state. When “grasp” or “shake” is selected, the motion is conducted, but the object is maintained. Because $w_{t}^{O}$ uses the information of the object being held, we set the object information to $w_{t-1}^{O}$, which is the information one step earlier. This process yields $w_{t}^{O}$ and $w_{t}^{M}$.

When the robot performs the above action selection, the human partner gives the verbal information and reward corresponding to the motion and the object. The reward *R*_*t*_ is given directly by key input as shown in Figure 6D for the object and motion. For verbal information, when the robot holds the ball and gets a positive reward, the human partner makes an utterance, such as “It's good to hold the ball, it's a soft ball.” Speech recognition is performed on these utterances to acquire sentences. We obtain $w_{t}^{w}$ by performing morphological analysis on these sentences with “MeCab,” which is a Japanese morphological analyzer. This yields $w_{t}^{w}$ and $w_{t}^{R}$.

By the above action, the robot gets multimodal information $w_{t}^{O}$, $w_{t}^{M}$, $w_{t}^{R}$, $w_{t}^{w}$. Based on this observed information, we calculate the top-level concept ${\bar{z}}_{t+1}$ of the next time step by policy function. This was done based on the Equations (3), (4), and (5). Depending on whether or not the object is held, either the object selection of the next state or the motion selection is performed by using the obtained top-level concept ${\bar{z}}_{t+1}$.

The experiment was performed with the above flow as one step and consisted of 50 steps per episode and 10 episodes, for a total of 500 steps. The parameters of the integrated cognitive architecture were updated online using multimodal information w* obtained for each episode. The aforementioned learned model parameters were stored as robot knowledge, and the robot used them to make action decisions and perform language understanding. The time required for the robot to make decisions at each step was 1 s. Therefore, the interaction between the robot and the user was sufficiently smooth. However, the learning process at each episode required 10 min; therefore, the user was required to wait for the robot to complete the learning process.

By repeating this, the robot learns concepts, actions, and language. At the 11th episode, action selection was performed using test data to verify the generalization of the model. Furthermore, using the data of 10 episodes and 500 steps acquired by the robot, two models with different structures are learned off-line. By analyzing the learned results off-line, we evaluate each module and verify the influence of the predictability by the integration of model modules.

## 5. Results

The integrated cognitive architecture was evaluated by analyzing the learning results. First, we show the results of concept formation and action learning. We then show the results of language learning. Finally, the influence of module integration is shown.

### 5.1. Concept Formation

Table 1 shows the accuracy of concept formation (categorization). The classification accuracy of Data 1 and 2 at training, and the classification accuracy for episode 11 using test data are shown for each low-level concept. Classification accuracy is calculated from the degree of agreement between the concept class estimated by the model and the ground truth shown in Figure 6. From this table, it is clear that all information can be classified correctly for the motion concept and the reward concept. As for the object concept, the classification accuracy is about 82% for the training data and 70% for the test data.

**Table 1:** Results of concept formation (accuracy).

	**Object**	**Motion**	**Reward**
Data1,2	0.818	1.00	1.00
Test data	0.700	1.00	1.00

### 5.2. Action Learning

**Figure 8:**
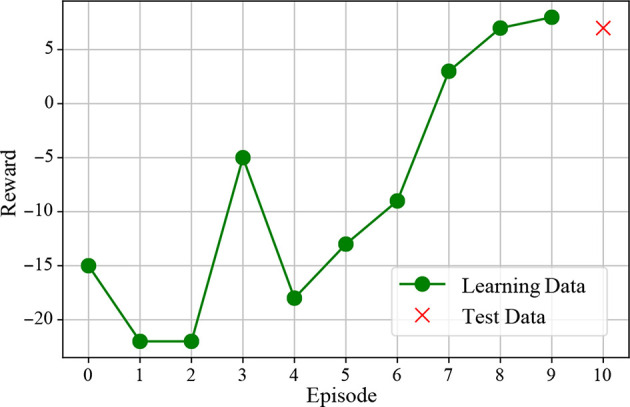
Accumulated reward for each episode. “x” represents the result for the test data, which is unknown for the robot.

The result of action learning is shown in Figure 8. This shows the accumulated reward for each episode. From this figure, it can be seen that the accumulated reward value increases as the number of episodes increases. In addition, the cumulative reward value similar to the learning data is obtained for the test data, which is a novel object, suggesting that the action selection with generalization is obtained.

### 5.3. Language Learning

**Figure 9:**
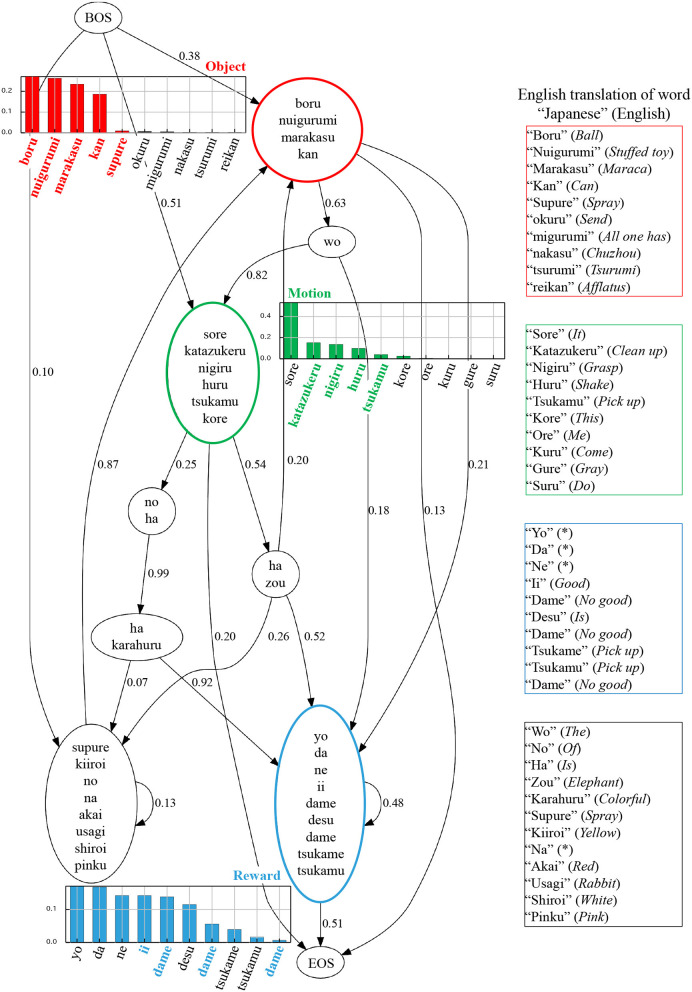
Visualization of learned language models. Each node represents a hidden state of BHMM, and an edge represents a transition probability. The words in the nodes represent the words corresponding to the hidden state. The red, green, and blue nodes are POS linked to the object, action and reward concepts, respectively. The bold colored characters represent words corresponding to the concepts annotated by humans. The * indicates that it can not be translated into English.

To analyze language learning, three tasks were performed: visualization of internal representation of the language model (BHMM), language understanding, and sentence generation. First, the internal representation of the learned language module is shown in Figure 9. Each node in Figure 9 represents a hidden state (POS) of BHMM, and an edge represents transition probability. The words in the node represent words corresponding to the POS. The bar graph in Figure 9 shows the output probability of the word corresponding to each low-level concept for the top 10 words. It is desirable that the nodes corresponding to each concept have a higher word output probability corresponding to each concept. From this figure, it can be seen that the words corresponding to each concept are connected to the correct concept. Moreover, the transition of each POS is nearly correct in terms of Japanese grammar. However, certain linguistic errors occurred. For example, the output probability of “sore”, which was not related to the motion, was the highest in the motion node. To verify language comprehension, we perform the task of predicting real-world information from linguistic information. Real-world information *w*^*O*^, *w*^*M*^, *w*^*R*^ was predicted from the 24 input sentences shown in Table 2. We calculated the degree of agreement between the predicted information and the information represented by the sentence. As a result, correct predictions were made for all sentences.

**Table 2:** Sentences used as inputs for the evaluation.

“Nuigurumi wo tsukame”	“marakasu wo tsukame”
(Pick up a stuffed toy)	(Pick up a maraca)
“Nuigurumi wo katazukeru no ha ii ne”	“marakasu wo katazukeru no ha ii ne”
(It is good to clean up stuffed toy)	(It is good to clean up maraca)
“Nuigurumi wo nigiru no ha ii ne”	“marakasu wo nigiru no ha ii ne”
(It is good to grasp stuffed toy)	(It is good to grasp maraca)
“nuigurumi wo nigiru no ha dame desu”	“marakasu wo nigiru no ha dame desu”
(It is not good to grasp stuffed toy)	(It is not good to grasp maraca)
“nuigurumi wo huru no ha ii ne”	“marakasu wo huru no ha ii ne”
(It is good to shake stuffed toy)	(It is good to shake maraca)
“nuigurumi wo huru no ha dame desu”	“marakasu wo huru no ha dame desu”
(It is not good to shake stuffed toy)	(It is not good to shake maraca)
“boru wo tsukame”	“supure kan wo tsukame”
(Pick up a ball)	(Pick up a spray can)
“boru wo katazukeru no ha ii ne”	“supure kan wo katazukeru no ha ii ne”
(It is good to clean up ball)	(It is good to clean up spray can)
“boru wo nigiru no ha ii ne”	“supure kan wo nigiru no ha ii ne”
(It is good to grasp ball)	(It is good to grasp spray can)
“boru wo nigiru no ha dame desu”	“supure kan wo nigiru no ha dame desu”
(It is not good to grasp ball)	(It is not good to grasp spray can)
“boru wo huru no ha ii ne”	“supure kan wo huru no ha ii ne”
(It is good to shake ball)	(It is good to shake spray can)
“boru wo huru no ha dame desu”	“supure kan wo huru no ha dame desu”
(It is not good to shake ball)	(It is not good to shake spray can)

**Figure 10:**
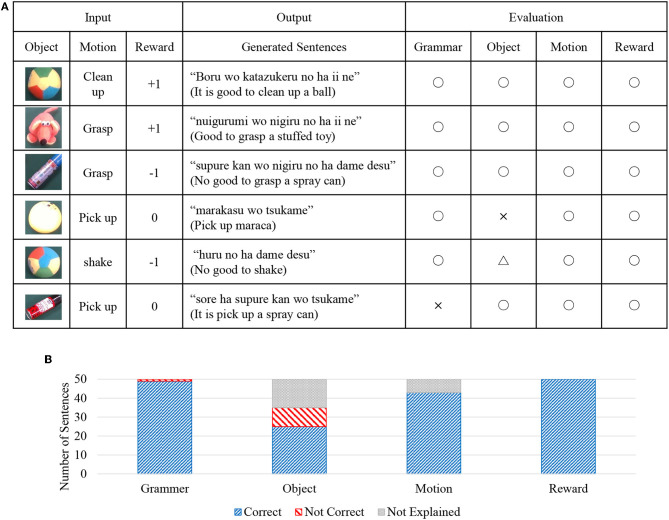
Results of sentence generation; **(A)** some examples and evaluations of generated sentences, and **(B)** evaluation results of generated sentences from four different criteria.

Sentences were generated using real-world information observed by the robot as input. By this evaluation, we verified that the robot could generate appropriate sentences. The sentences were generated by the method shown in Figure 5D. Input data was generated for the input of each step, using real-world data *w*^*O*^, *w*^*M*^, *w*^*R*^ for 50 steps of episode 11, which is the test data. The result is shown in Figure 10. Figure 10A shows the result of 6 steps out of 50 steps. The input values are the real-world information observed by the robot at each step. The evaluation of the sentence is performed to determine if the generated sentence is grammatically correct (○ or ×) or whether the explanation in the language is correct (○ or ×) for the information of the object, action, and reward. In addition, the case in which the information is not explained is marked as (△).

For example, because correct sentences are generated for each observed information from the top three in Figure 10A, all evaluations are all correct (○). The fourth result is a mistake (×) for the object information, because the sentence of “pick up maraca” is generated, although the observed object is a ball. The fifth result is an evaluation of no explanation (△), because the object is not explained in sentences. The final result is that the explanation is correct, but because it is incorrect in terms of Japanese grammar, it is evaluated that the grammar is incorrect (×).

A summary of these results for all generated sentences (50 sentences) is shown in Figure 10B. It can be seen from this figure that, except for the object, nearly correct sentences are generated.

### 5.4. Effects on Integration of Modules

Two models were compared to verify the impact of module integration. One is the proposed integrated model using the module shown in Figure 4. The second is a model obtained by removing the language module (BHMM) from the first model. In other words, the second model is a model in which a lexicon exists but a syntax module does not exist. We label these model 1 and model 2, respectively. In model 2, because BHMM is missing, word information *w*^*C*^ for each concept is not weighted by the language model, and all *w*^*wC*^ is treated as *w*^*w*^ at the learning and recognition stages.

**Figure 11:**
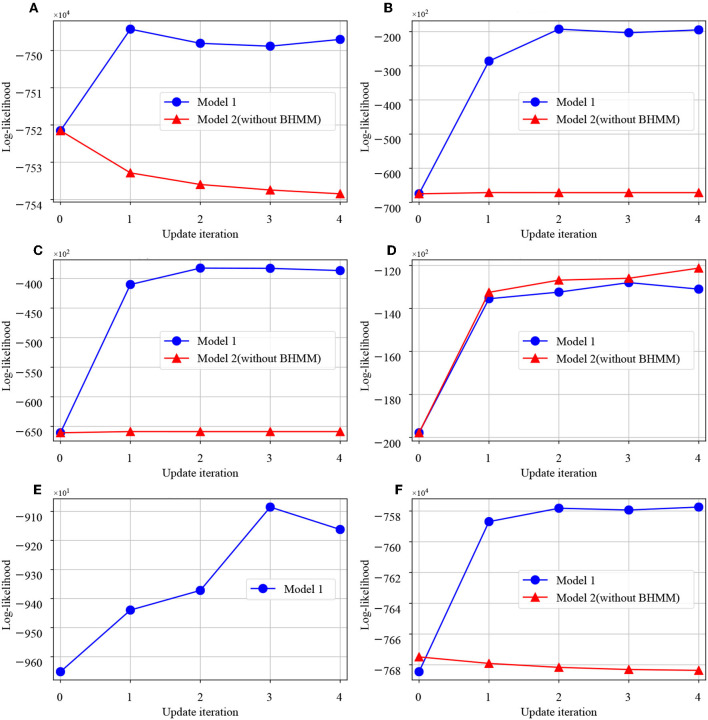
Graphs of log-likelihood change in each module. The horizontal axis shows the number of updates of the whole model, and the vertical axis shows the log-likelihood of each module. **(A)** MLDA-object, **(B)** MLDA-motion, **(C)** MLDA-reward, **(D)** MLDA-top, **(E)** BHMM, **(F)** All-module.

First, we show changes in log-likelihood during the training of model 1 and model 2. Figure 11 shows the change in the log-likelihood of each module during learning. The vertical axis indicates the log-likelihood, and the horizontal axis is the number of learning iterations of the model. From these figures, it can be seen that the log-likelihood of the low-level concept is increased in model 1, including the language module. Also, with regard to the top-level concept, the log-likelihood is increased as mutual learning progresses in both models.

**Figure 12:**
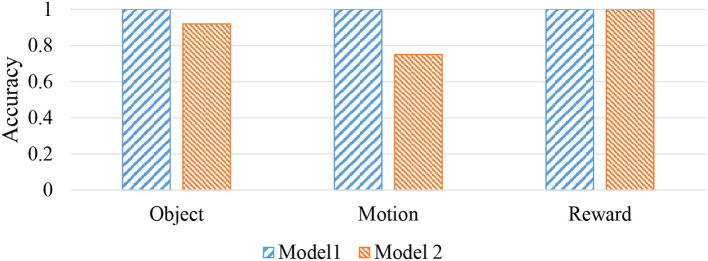
Prediction accuracy of real-world information from linguistic information.

To verify the change in predictability due to the presence or absence of the language module, the task of predicting real-world information from language information was also performed for model 2. The result is shown in Figure 12. From this result, as described above, model 1 can make correct predictions for all sentences. The model 2 shows a drop in prediction accuracy for objects and motions.

**Figure 13:**
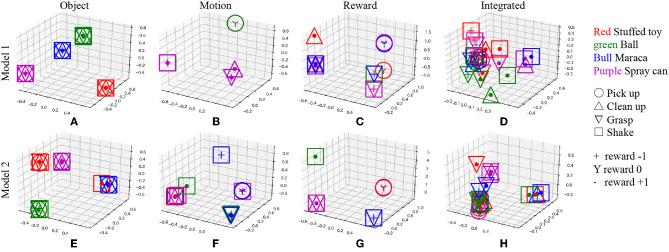
Visualization of conceptual space of each MLDA inferred from sentences; **(A)** object concept of model 1, **(B)** motion concept of model 1, **(C)** reward concept of model 1, **(D)** integrated concept of model 1, **(E)** object concept of model 2, **(F)** motion concept of model 2, **(G)** reward concept of model 2, and **(H)** integrated concept of model 2. The legend indicates the category of each concept corresponding to the input sentence.

The concept space z* estimated from each sentence was compressed and visualized in three-dimensional space using principal component analysis (PCA) to analyze the details of the above results. Figure 13 shows the result for each conceptual space of each model. In model 1, with high prediction accuracy of real-world information, sentences are well-categorized according to the type of each concept. On the other hand, the result of model 2 shows that confusion occurs in the conceptual space with respect to the information pointed by the sentence in the object concept and the motion concept.

## 6. Discussions

The purpose of this study is to examine the acquisition of multiple cognitive functions such as concept formation, decision making, and language learning by robots through the integration of multiple cognitive modules, centered on mMLDA, as well as to verify the effect on integration of multiple modules.

First, we discuss the decision making. Figure 8 shows the increase in the accumulated reward value, and it is suggested that appropriate action learning is possible by trial and error in the real environment. The action selection is performed by using the formed concept as a state-action space, as shown in Figure 5B. In other words, it is possible to compress information by structuring multimodal information with mMDLA and use it for decision making. Furthermore, since the object concept and the motion concept can be generated from the top-level concept, the object and action selection can be made by determining the top-level concept.

Next, we will describe the internal representation of the language model. In Figure 9, certain linguistic errors occur. In the motion node, the word “sore” (“it” in English), which does not relate to the motion concept directly, has the highest output probability. We hypothesize that this error occurred owing to the effect of word order. It was observed that the input sentences contained sentences with similar structure, such as “sore ha ii ne” and “(Japanese verb) no ha ii ne” (“it is good” and “(verb) is good” in English, respectively) with high frequency. It may be observed that in these patterns, there is a strong connection between the verb and “sore” (“it”), which resulted in the assignment of the word “sore” (“it”) to the motion node. In the reward node, the Japanese particles “da”, “yo”, and “ne” were incorrectly connected to the reward concept. We assume that this occurred because these particles often co-occurred with reward words, such as “good” or “bad”. We also presume that these errors can be corrected gradually by learning continuously using more diverse data.

Next, language understanding is described. In language understanding by robots, it is important to determine how accurately real-world information can be recalled from linguistic information. Figure 12 shows that high prediction accuracy is achieved in the prediction task from input sentences. It is also interesting that language learning makes it possible to account in unseen situations. For example, the sentence “good to shake stuffed animal” is not actually observed because it violates the experimental setting. However, even for such sentences, information on each object, action, and reward can be correctly predicted. This is one of the important features of the language that one can recall what has never been observed.

Then, we will describe sentence generation. Figure 10 shows that most of the generated sentences are grammatically correct and can handle syntactic information correctly. As for object information, 25 out of 50 sentences were correct, and 10 sentences were incorrect. Ten sentences that have been mistaken are generated as sentences in which the ball category is confused with maracas and stuffed animals. These are the results that appeared in the sentence generation demonstrating the effect of being unable to form the object concept well. In terms of motion information, 43 sentences out of 50 showed correct language information. In the seven sentences that did not show correct linguistic information, the action of grasping was selected while the object being held. In this case, because the robot does not move (because it cannot hold the object from the object-holding state), the user did not give verbal information for such a situation during the experiment. For this reason, it is thought that a sentence that explains motion information has not been generated.

These cognitive functions are realized by the integration of models, which form appropriate concepts (latent space). In other words, by integrating modules through hidden variables, multiple cognitive functions can share knowledge and realize various tasks.

Next, the influence of module integration is discussed. Figure 11 shows the log likelihood during model learning. From this figure, it can be seen that the log likelihood increases with model 1 in which the language modules are integrated, rather than model 2 in which language modules are missing, as the update of the entire model proceeds. In addition, in the task of language understanding to predict real-world information from input sentences, model 1 with integrated language modules showed higher prediction performance than model 2. This is considered to be the optimization of the model as a whole and the improvement of the prediction performance because the word information is updated to one suitable for each concept by the integration of modules. There are three main modules in the proposed architecture, namely the language, action, and concept-formation modules. However, in this study, we verified the relationship between language and concept formation by removing the language module. We only verified the effect of the integration of the language module because it was relatively easy to make a fair comparison. For example, if the action module would be removed from the proposed model, it would be difficult to compare with- and without-action modules because of the acquired data changes. In addition, if the mMLDA was missing, the core module would no longer exist. In this case, the updating rule of the proposed model cannot be applied. Owing to these issues, we examined the effect of integration regarding the language module only.

Finally, we will describe the reasons for the selection of each module. As a major premise, we considered the candidate model that would be able to connect other modules through the SERKET framework to render the entire model tractable. Within this constraint, we selected each module. The purpose of selecting the mMLDA as a concept-formation (unsupervised learning) algorithm has been described in section 2.2. The BHMM was selected for language learning because our previous work revealed the advantages of the mMLDA and the BHMM in simultaneous learning in terms of language and concepts. Regarding reinforcement learning, in this study, the module must be able to operate in the continuous state space. Hence, we selected the REINFORCE algorithm, which is one of the simplest algorithms that operates in the continuous state space. In this study, we selected the aforementioned algorithms; however, the proposed framework allows the substitution of current modules with other modules that have similar functionalities. For example, the mMLDA can be replaced with a neural topic model based on the variational autoencoder (Miao et al., 2016; Srivastava and Sutton, 2017). In language learning, long-term and short-term memory (LSTM) (Zaheer et al., 2017), as well as the bidirectional encoder representations from transformers (BERT) (Devlin et al., 2018), may be used. In reinforcement learning, several models exist, such as the soft actor critic (SAC) (Haarnoja et al., 2018), that could replace REINFORCE algorithms. In our future work, we consider employing the aforementioned models because the performance of the cognitive architecture can be improved.

## 7. Conclusions

In this paper, we realized an integrated cognitive architecture for learning concepts, actions, and language by integrating multiple probabilistic generative models. To verify the validity of the proposed model, an experiment involving the interaction between a real robot and a human partner in a real physical environment was conducted. Through the experiment, the robot acquired multimodal information and learned actions and language. The effectiveness of the proposed model was verified by analyzing the learned model by the robot. As a result, the robot learned concepts, actions, and language based on its own experiences. By using the learned model, various cognitive functions such as action selection through latent variables, prediction of real-world information from language, and generation of sentences become possible. In addition, we examined the change in the prediction performance by having integrated several modules. Specifically, we confirmed that the performance of the prediction improves when language modules are integrated. A framework in which a robot learns by integrating multiple cognitive modules is important for the construction of human-like intelligence. It is fair to say that this research, in which concepts, actions, and language are simultaneously learned by robots, has provided insight toward the achievement of that ultimate goal. In the future, enabling higher-level inference, e.g., planning, will be addressed by explicit modeling of temporal information. In addition, it is necessary to further verify the integrated model by performing a task that drives a plurality of modules simultaneously.

## Data Availability Statement

The datasets generated for this study are available on request to the corresponding author.

## Author Contributions

KM, TH, TA, and TN conceived of the presented idea. KM and TN developed the theory. KM implemented the system and conducted the experiment. KM wrote the manuscript with support from TN, TH, and TA. All authors discussed the results and contributed to the final manuscript.

### Conflict of Interest

The authors declare that the research was conducted in the absence of any commercial or financial relationships that could be construed as a potential conflict of interest.
