# Supplementary material for: Integrated Cognitive Architecture for Robot Learning of Action and Language
Source: Front Robot AI. 2019 Nov 29;6:131. doi: 10.3389/frobt.2019.00131 (PMC7805838; doi:10.3389/frobt.2019.00131)
Supplement: Supplementary file 1 [file Data_Sheet_1.pdf]

APPENDIX A: INFERENCE OF MLDA

In this appendix, the update rule of MLDA is explained. MLDA can be optimized through the parameters θ and ϕ by inferring the concept z using Gibbs sampling. Gibbs sampling of concept z is carried out by the following formula.

$$P(z_{mij} = k|\mathbf{W}, \mathbf{Z}^{\backslash mij}, \alpha, \beta^m) \propto (n_{k,j}^{\backslash mij} + \alpha)\frac{(n_{m,w^m,k}^{\backslash mij} + \beta^m)}{(n_{m,k}^{\backslash mij} + W^m\beta^m)}, \tag{A-1}$$

where W^m and $\mathbf{W}$ denote the number of dimensions of the m-th modality and the observed multimodal information, respectively. $n_{m,w^m,k,j}$ represents the number of times the j-th data modality m is w^m and category k has been allocated. The subscript ”\” in Eq.(A-1) indicates that the information is excluded. In other words, $\mathbf{Z}^{\backslash mij}$ is the remainder set of concepts after removing category z_{mij} assigned to the i-th information of the modality m of the j-th data. According to the above equation, the category is assigned to the i-th information of the modality m in j-th data. Repeat this until n_* converges. From the finally converged values, the model parameters θ^* and ϕ^* are obtained as follows:

$$\theta_{kj} = \frac{n_{k,j} + \alpha}{n_j + K\alpha}, \tag{A-2}$$

$$\phi_{w^m,k}^m = \frac{n_{m,w^m,k} + \beta^m}{n_{m,k} + W^m\beta^m}, \tag{A-3}$$

where K is the total number of categories.

APPENDIX B: PREDICTION IN MLDA

This appendix describes prediction of category for novel input data using a learned MLDA model. The following equation estimates the concept $\hat{z}$ in the observed new data $\boldsymbol{w}_{obs}$.

$$\hat{z} \sim P(z|\boldsymbol{w}_{obs}) = \int P(z|\theta)P(\theta|\boldsymbol{w}_{obs})d\theta, \tag{A-4}$$

where $P(\theta|\boldsymbol{w}_{obs})$ is obtained by recalculating θ while fixing the parameters estimated during the learning and applying the Gibbs sampling described above. The category is estimated by selecting the category k that maximizes the concept probability as;

$$k = \underset{z}{\operatorname{argmax}}\, P(z|\boldsymbol{w}_{obs}). \tag{A-5}$$

By using the learned model, it is possible to predict unobserved information. The unobserved information w is estimated from the observed information $\boldsymbol{w}_{obs}$ as

$$P(w|\boldsymbol{w}_{obs}) = \int \sum_z P(w|z)P(z|\theta)P(\theta|\boldsymbol{w}_{obs})d\theta, \tag{A-6}$$

where $P(\theta|\boldsymbol{w}_{obs})$ is obtained by recalculating θ applying Gibbs sampling described above with the parameters estimated during learning process.

APPENDIX C: LANGUAGE LEARNING

We explain how to realize language learning in this appendix. First, the sentences are divided into words, and a multimodal categorization is conducted, using mMLDA, on the assumption that all words belong to all concept classes. We then update mMLDA and computing the association between words w and category k of concept $C \in \{Object, Motion, Reward\}$ using mutual information. The mutual information is calculated as,

$$I(w;k|C) = \sum_{K,W} P(W,K|C) \log \frac{P(W,K|C)}{P(W|C)P(K|C)}, \quad for\ K \in (k,\bar{k})\ \ and\ \ W \in (w,\bar{w}), \tag{A-7}$$

where $\bar{k}$ represents all categories excluding k, and $\bar{w}$ represents the words except w. Words with large amounts of mutual information are considered to be related to the concept, and the words with small amounts of mutual information are considered to be functional words. By this calculation, the robot can find out the relationship between words and POS $s \in \{C, functional\}$ including concept classes C and functional words ($functional$). The word information w^{wC} can be estimated from real-world observation $\boldsymbol{w}_{obs} \in \{w^O, w^M, w^R\}$ as

$$P(w^{wC}|\boldsymbol{w}_{obs}, s) \propto \max_k P(w^{wC}|s)P(w^{wC}|k)P(k|\boldsymbol{w}_{obs}, s), \tag{A-8}$$

where $P(w^{wC}|k)$ and $P(k|\boldsymbol{w}_{obs}, s)$ can be calculated using MLDA. It should be noted, for functional words, $P(w^{wC}|k)$ and $P(k|\boldsymbol{w}_{obs}, s)$ are treated as uniform distribution, since the relationship with the concept is small. $P(w^{wC}|s)$ is the output probability ξ of the word from POS, and the learning method is explained next.

We use the mutual information as the BHMM's initial value and estimated POS using the BHMM. By this, the concept formation of mMLDA affects the learning of the BHMM. The transition probability ρ and the output probability ξ of the BHMM parameters are shown below.

$$\rho_{b,b-1} = P(s_b|s_{b-1}) = \frac{n_{s_{b-1},s_b} + \gamma}{\sum_{s_b} n_{s_{b-1},s_b} + N_s\gamma}, \tag{A-9}$$

$$\xi_{w_b,s_b} = P(w_b|s_b) = \frac{n_{s_b,w_b} + \mu}{\sum_{w_b} n_{s_b,w_b} + N_w\mu}, \tag{A-10}$$

where n_{s_{b-1},s_b} and n_{s_b,w_b} are the number of transitions from s_{b-1} to s_b, and the number of times the word w_b has been output from s_b, respectively, N_s and N_w are the total number of POS and total number of words, respectively, and γ and μ are hyperparameters of the BHMM. The parameters of the BHMM are updated by Gibbs sampling from the following equation,

$$P(s_b = s|w_b, s_{b+1}, s_{b-1}) \propto P(s_b = s|s_{b-1})P(s_{b+1}|s_b = s)P(w_b|s_b = s). \tag{A-11}$$

As we mentioned earlier, the BHMM estimates the POS. The result of concept selection for each word through the mMLDA is used as the initial guess of the BHMM. Here, grammar is represented as the transition probability among the concept classes in the learned BHMM. It should be noted that the number of classes must be determined manually in advance.

By updating the BHMM, the word output probability $P(w^w|s)$ from each POS s considering syntactic information can be obtained. By using this as a bias, word information w^{wC} corresponding to each concept

C is found from observation w^w;

$$w^{wC} \propto w^w P(w^w|s). \tag{A-12}$$

Using the word information w^{wC} obtained by the above equation as the word input for each concept of mMLDA, the mMDLA is updated again. By iterating the above procedure several times, the robot can acquire the POS (i.e., the connection of concept class and word), grammar, and concepts.

APPENDIX D: SENTENCE GENERATION

By using the learned model, sentences can be generated from observation $\boldsymbol{w}_{obs} = \{w^O, w^M, w^R\}$. This appendix shows the algorithm that generates a sentence using observations.

First, N concept class sequences from begin of sentence "BOS" to end of sentence "EOS" are sampled according to Eq.(A-9). Let $\boldsymbol{s}^n = \{s_1^n, \cdots, s_t^n, \cdots, s_{T_n}^n\}$ be the n-th sample excluding "BOS" and "EOS", where T_n represents the length of the concept class sequence and corresponds to the length of the sentence. Then, from the POS s_t^n, the word corresponding to the concept class is estimated according to Eq.(A-8). Here, for given observation $\boldsymbol{w}_{obs} \in \{w^O, w^M, w^R\}$, the top K words with high probability $\boldsymbol{w}_t^n = \{w_{t1}^n, w_{t2}^n, \cdots, w_{tK}^n\}$, corresponding to the POS s_t^n are selected, and the set of all the words is represented by $\boldsymbol{W}^n = \{\boldsymbol{w}_1^n, \boldsymbol{w}_2^n, \cdots, \boldsymbol{w}_{T_n}^n\}$. That is, K^{T_n} patterns of sentences can be generated from these concept sequences and words, and the probability of sentence S^n is defined as follows;

$$P(S^n|\boldsymbol{s}^n, \boldsymbol{W}^n, \boldsymbol{w}_{obs}) \propto \prod_b P(s_b^n|s_{b-1}^n) P(w_b^n|\boldsymbol{w}_{obs}, s_b^n) P(w_b^n|w_{b-1}^n), \tag{A-13}$$

where $P(w_b^n|w_{b-1}^n)$ represents the word bigram and can be calculated as

$$P(w_b|w_{b-1}) = \frac{n_{w_{b-1},w_b} + \epsilon}{\sum_{w_b} n_{w_{b-1},w_b} + N_w \times \epsilon}. \tag{A-14}$$

b and n_{w_{b-1},w_b} represent the index of order in words, and the frequency of the occurrence of w_{b-1} to w_b consecutively, and ϵ is a coefficient to be determined in advance. Note, $P(w_b^n|\boldsymbol{w}_{obs}, s_b^n)$ in Eq.(A-13) is calculated by Eq.(A-8).

From the sentences generated from N concept sequences and words sampled for a given observation, the sentence with the highest probability is selected. First, from each concept sequence, the sentence $\hat{S}^n$ that maximizes Eq.(A-13) is searched using Viterbi algorithm. Here, let $\hat{\boldsymbol{S}} = \{\hat{S}^1, \cdots, \hat{S}^n, \cdots, \hat{S}^N\}$ be the set of sentences with the highest probability for each of N concept class sequences. Finally, the sentence with the highest probability is selected from $\hat{\boldsymbol{S}}$. Because the longer the sentence is, the lower the probability is, the following adjustment factor $\ell(\hat{S}^n)$ is introduced.

$$\ell(\hat{S}^n) = \frac{(L^{\max} - L_{\hat{S}^n})}{\sum_n^N L_{\hat{S}^n}} \sum_n^N \log P(\hat{S}^n|\boldsymbol{s}^n, \boldsymbol{W}^n, \boldsymbol{w}_{obs}), \tag{A-15}$$

where $L_{\hat{S}^n}$ and $L^{\max}$ represent the length of the sentence $\hat{S}^n$, and the maximum length of the sentence in $\hat{\boldsymbol{S}}$. Using Eq.(A-15), the score of the sentence is redefined as

$$\log \bar{P}(\hat{S}^n|\boldsymbol{s}^n, \boldsymbol{W}^n, \boldsymbol{w}_{obs}) = \log P(\hat{S}^n|\boldsymbol{s}^n, \boldsymbol{W}^n, \boldsymbol{w}_{obs}) + \omega\ell(\hat{S}^n), \tag{A-16}$$

where ω is a weight to adjust the length of the sentence. The larger the weight is, the longer the sentence is. Therefore, final sentence S is obtained by

$$S = \underset{\hat{S}^n \in \hat{\boldsymbol{S}}}{\operatorname{argmax}} \log \bar{P}(\hat{S}^n | \boldsymbol{s}^n, \boldsymbol{W}^n, \boldsymbol{w}_{obs}). \tag{A-17}$$
